# Authors’ attitude toward adopting a new workflow to improve the computability of phenotype publications

**DOI:** 10.1093/database/baac001

**Published:** 2022-02-02

**Authors:** Hong Cui, Bruce Ford, Julian Starr, Anton Reznicek, Limin Zhang, James A Macklin

**Affiliations:** School of Information, University of Arizona, 1103 E. Second Street, Tucson, AZ 85705, USA; Department of Biological Sciences, University of Manitoba, 50 Sifton Road, Winnipeg, MB R3T 2N2, Canada; Department of Biology, University of Ottawa, 30 Marie Curie Road, Ottawa, ON K1N 6N5, Canada; SLA Herbarium, University of Michigan, 3600 Varsity Drive #1046, Ann Arbor, MI 48019, USA; School of Information, University of Arizona, 1103 E. Second Street, Tucson, AZ 85705, USA; Ottawa Research and Development Centre, Agriculture and Agri-Food Canada, 960 Carling Avenue, Ottawa, ON K1A 0C6, Canada

## Abstract

Critical to answering large-scale questions in biology is the integration of knowledge from different disciplines into a coherent, computable whole. Controlled vocabularies such as ontologies represent a clear path toward this goal. Using survey questionnaires, we examined the attitudes of biologists toward adopting controlled vocabularies in phenotype publications. Our questions cover current experience and overall attitude with controlled vocabularies, the awareness of the issues around ambiguity and inconsistency in phenotype descriptions and post-publication professional data curation, the preferred solutions and the effort and desired rewards for adopting a new authoring workflow. Results suggest that although the existence of controlled vocabularies is widespread, their use is not common. A majority of respondents (74%) are frustrated with ambiguity in phenotypic descriptions, and there is a strong agreement (mean agreement score 4.21 out of 5) that author curation would better reflect the original meaning of phenotype data. Moreover, the vast majority (85%) of researchers would try a new authoring workflow if resultant data were more consistent and less ambiguous. Even more respondents (93%) suggested that they would try and possibly adopt a new authoring workflow if it required 5% additional effort as compared to normal, but higher rates resulted in a steep decline in likely adoption rates. Among the four different types of rewards, two types of citations were the most desired incentives for authors to produce computable data. Overall, our results suggest the adoption of a new authoring workflow would be accelerated by a user-friendly and efficient software-authoring tool, an increased awareness of the challenges text ambiguity creates for external curators and an elevated appreciation of the benefits of controlled vocabularies.

## Introduction

In order to answer large-scale biological questions, knowledge produced by different biological branches needs to be integrated. For that purpose, a large number of ontologies have been developed (e.g. see the OBO Foundry (1)). Ontologies hold established biological terms and the relationships among the concepts represented by these terms, and they can be seen as an advanced form of controlled vocabularies. Many of these ontologies are being used in biological data curation to convert published phenotype knowledge (including taxonomic descriptions and character descriptions) from their current narrative format to an ontologized format (i.e. computable format, a.k.a., a machine-actionable format that can be used in computational analyses (2, 3)). In this process, data curators translate the author’s terminologies used in publications to the standardized terms included in the ontologies. Computable phenotypes are critical data not only for taxonomic research but also for various branches of evolutionary biology and ecology (3–5).

There is more published phenotypic information than professional curators can process (2, 6). Taking into consideration the continued publication of such information in massive amounts in the traditional format, generating FAIR data (Findable, Accessible, Interoperable and Reusable (7),) through professional curation faces a significant obstacle. On top of workload issues, inter-curator variation (Inter-curator variation refers to the phenomenon where the curated results from multiple curators on the same source data are different) is also a main concern, as within-project variation has been reported to be as high as 40% or greater in multiple studies (8–10). Integrating data from multiple projects over time can be expected to amplify the level of variation. Inter-curator variation is largely caused by inconsistent usages of ambiguous terms in published phenotype descriptions (8).

We have been funded by the US National Science Foundation to renew the investigation of an approach where authors produce computable data at the time of publication to reduce curation costs and to avoid inter-curator and curator-author variation. This approach is not entirely new: back in 1990s, when the Flora of North America Association started to produce taxonomic accounts of all the plants species occurring north of the Mexican border, they asked authors to enter taxonomic descriptions in a structured form using a controlled vocabulary (i.e. (11)) to promote consistency and reduce ambiguity. The authors resisted, and the plan was soon abandoned (Pers. Comm. With Dr Bruce Ford February, 2021, Dr James Macklin, November 2015, Dr Bryan Heidorn May, 2000). In our proposal, we argued that science authors should be provided with tools to enable them to produce computable data directly while they write for their human audience, as opposed to relying upon the costly, variation-prone, post-publication curation processes. We also argued against using a controlled vocabulary that limits the ability of authors to construct complex taxonomic descriptions. In this project, we develop and evaluate the effects and usability of several new ontology-powered data recording tools that promote clarity and parallelism in taxonomic descriptions and allow authors to contribute and improve upon existing ontologies. As part of the investigation, we also conducted a survey on the attitude of biologists toward using controlled vocabularies/ontologies to make phenotypic data computable. The finding informed the design of the software tools. The questions we sought to answer through the survey included:

1. What are the respondents’ current experience and overall attitude with controlled vocabularies?
2. What is the respondents’ awareness of data quality and curation issues? In this paper, ‘data quality issues’ refers to ambiguous terminology and inconsistent term usage.
3. What are the respondents’ attitude and current actions toward data quality issues?
4. What is the respondents’ preference toward candidate solutions?
5. What levels of effort are the respondents willing to make in order to adopt new solutions, and what are their desired rewards?

In this paper, we describe the development of the survey and report the answers to the above questions. These answers shed light on the factors that contribute to respondents’ willingness to take action or their resistances to proposed changes.

## Materials and methods

### Development of survey questions

Attitude is ‘a relatively enduring organization of beliefs, feelings, and behavioral tendencies towards socially significant objects, groups, events or symbols’ (12) and ‘a psychological tendency that is expressed by evaluating a particular entity with some degree of favor or disfavor’ (13).

Among various models of attitude, the ABC model is one of the most cited (14). The ABC model suggests that any attitude consists of three components: an affective component, a behavioral component and a cognitive component. While every attitude is a manifestation of all three components, any particular attitude can be based on one component more than another. These components help to explain where an attribute comes from. The affective component refers to an individual’s feelings/emotions toward an attitude object (such as a person, a thing or an event). The behavior component denotes the way people behave when exposed to an attitude object. A certain behavior reflects one’s attitude toward the attitude object, while a certain attitude could also be the cause of certain behaviors. The cognitive component involves an individual’s belief or knowledge about the attitude object. For example, an individual with substantial knowledge of how ontologies have helped improve data management efforts may have a strong positive attitude toward a new ontology. This attitude is largely based on the cognitive component. If we observe, this individual consistently uses this ontology and provides positive comments on it, this behavior reflects the person’s positive attitude toward the attitude object.

The ABC model was followed in designing the questionnaire. We first drafted groups of questions corresponding to the affective, behavioral and cognitive aspects of attitude, then rearranged the questions so that they follow a logical order, largely corresponding to the research questions listed above. Co-authors read the questions to improve their readability. Questions with any ambiguities identified were rewritten until they were clear.

The vast majority of the questions in the survey are represented with a question statement and a set of selections presented on a 5-point Likert scale, i.e. strongly disagree, somewhat agree, neither agree nor disagree, somewhat agree and strongly agree, which is commonly used for individual opinions and judgments (15). Questions included in the survey are presented in Results.

### Survey mechanism and distribution

In this study, two versions of the survey were deployed on Qualtrics, an online survey tool (https://www.qualtrics.com/).

The initial (or Version 1) survey did not include any question on the field of study of the respondents. The link to this version was distributed through a number of mailing lists and informal professional networks from November 2018 to February 2019. The mailing lists included Biological Systematics Discussion List (Taxacom, November 2018), iDigBio-L (November 2018), Natural History Collections Listserver (Nncoll-l, November 2018), American Society of Plant Taxonomists (November 2018), the Society of Herbarium Curators (November 2018) and the Canadian Botanical Association’s Systematics & Phytogeography Section (February 2019). These societies represent a broad group of professional biologists with expertise in botany, zoology and the curation of biological collections.

Upon reviewing the responses from the Version 1 survey, some authors suggested that the knowledge and use of controlled vocabularies could be more developed in zoology than in botany, thus creating a concern that gathered responses might reflect a perspective shared by zoologists that is not shared by botanists. Out of this concern, a Version 2 of the survey was produced and distributed to the Botanical Society of America (July 2019), the largest botanical society in North America, and again to Taxacom (December 2019). This version of the survey remains accessible at https://uarizona.co1.qualtrics.com/jfe/form/SV_6VRPiQFGFNzYCwd. The last response included in this analysis was dated 9 December 2019.

In both versions, there was a section called ‘Background and Terminology’ (see Supplementary Appendix B) where we defined an ontology as [a knowledge structure] that holds established terms and relationships among these terms. We explicitly stated that ontologies can be seen as an advanced from of controlled vocabularies.

The questions for Version 2 were similar to Version 1, with the exception that a Yes/No question was added after the Background and Terminology section to ask if the respondent was familiar with terms such as ‘ontologies’, ‘controlled vocabularies’ and ‘data curators’. The No choice explicitly noted that the level of familiarity with these terms did not affect the respondent’s ability to answer this attitude survey. A second question was also added in Version 2 to ask if respondents’ research or data work was mostly in the zoology or botany areas, neither, or both. The two questions that asked respondents to enter their job title and to list the controlled vocabularies they are aware of were removed from Version 2, to keep the number of questions the same across the two versions.

Administrating Version 2 of the survey allowed us to compare two different groups, defined here as ‘Biology respondents’ and ‘Botany Respondents’. We were able obtain a better idea about general attitudes toward using controlled vocabularies/ontologies to make phenotypic data computable and whether Botany Respondents hold significantly different attitudes than others.

The survey (both versions) was anonymous, although respondents wishing to enter a $50 Amazon Gift Card draw provided an email address. Respondents were essentially self-selected, as they could leave the survey or skip questions at any time.

### Analysis methods

In addition to descriptive statistics (e.g. count, mean and standard deviation), a set of correlation analyses were also conducted to reveal any associations among the answers to different questions. When the variables are ordinal (e.g. Table 1, Q5), Spearman’s rank correlation tests were performed. When the variables are nominal (Table 1, Q6.v2.2), Fisher’s exact tests for independence were applied.

**Table 1.** Questions related to demographic information

QID	Questions	Options
Q2^b^	Your job title	Text input box
Q4	The length of your professional experience	<1 year; 1–5 years; 6–10 years; 10–15 years; more than 15 years
Q5	Your education level	BS/BA; MS/MA; Ph.D.; others
Q6v2.2^a^	Your research or data work is mostly in	Zoology areas; Botany areas; Neither; Both
Q7	Does your job involve (1) phenotype information/data creation or production (2) phenotype information/data management (3) phenotype information/data use or analysis (4) collection management (e.g. specimen collections) (5) research and publication (6) interdisciplinary/cross-disciplinary activities	Rarely, less than 5% of my work; Occasionally, in about 25%; Sometimes, in about 50%; Frequently, in about 75%; Usually, in about 95%;

a Added question in Version 2.

b Question removed from Version 2.

We coded the ordinal data in the following way: ‘strongly disagree’ on a 5-point Likert scale is coded as 1, while ‘strongly agree’ as 5. Answers that present a clear logic order are also coded as ordinal data using the principle of the stronger the answer, the greater the order. For example, for Q5 (Table 1), ‘less than one year’ is coded as 1, while ‘more than 15 years’ is coded as 5. For Q9 (Table 2), ‘never heard of it’ is coded as 1, while ‘Created it’ is coded as 4. For Q15 (Table 3), ‘false’ is coded as 1, and ‘true’ is coded as 2. The exact coding for each question can be seen in the figures presented in the Results section.

**Table 2.** Questions related to current experience and overall attitude toward controlled vocabularies

QID	Questions	Options
Q3v2.1^a^	[C] I am familiar with the bold terminology mentioned above.	Yes; No
Q8	[C] By your assessment, how many of your colleagues (1) know about controlled vocabularies, (2) use controlled vocabularies in their job, (3) use controlled vocabularies in their publications.	Very few; Some; Many
Q8vI^b^	[C] List examples of controlled vocabularies you know or have heard of (if any):	Text input box
Q9	[C] Your knowledge or experience of controlled vocabulary can be described as:	Never heard of it; Know the concept; Used it; Created it
Q28	[T] My overall attitude toward controlled vocabularies is	Negative; Neutral; Positive

a Added question in Version 2. This question appears after a paragraph of text, including terms such as ‘ontology’ and ‘curation’, which are highlighted in bold. See ‘Version 2: Background and Terminology’ in Supplementary Appendix B.

b Question in Version 1 but removed from Version 2.

**Table 3.** Questions related to the awareness of data quality and curation issues

QID	Questions	Options
Q10	[A] I feel frustrated by ambiguous phenotypic terms or measurements seen in some biology publications.	5-point scale
Q11	[C] To make phenotype information in research articles useful for computers, the information needs to be formatted in a computer-accepted manner.	5-point scale
Q13	[C] Most biologists lack the skills to convert human-readable phenotype information to a computable format.	5-point scale
Q14	[C] I am aware that when curators convert phenotypic characters to a computable format using controlled vocabularies, there can be as high as 40% variation in the results produced by different curators. The variation can be attributed to the lack of good matching terms in controlled vocabularies and to the vagueness in the character descriptions.	True/False
Q15	[B] If I know a phenotypic character in my publication was curated into a computable format, but the new format does not convey the original meaning of the character, I will attempt to have it corrected.	5-point scale
Q16	[C] In my opinion, when converting a phenotype character to a computable format, it is the authors, rather than data curators, who are more capable of retaining the original meaning of the character.	5-point scale

**Table 4.** Questions related to attitude and current actions to solve data quality issues

QID	Questions	Options
Q12	[A] I appreciate the work of individuals (curators) who convert phenotype information into a computable format.	5-point scale
Q17	[B] I would not make an effort to use any controlled vocabularies in my publication if it is not mandatory.	5-point scale
Q19	[B] I do not care what phenotype terms my colleagues have used to describe a character I am describing.	5-point scale
Q22	[B] I do not care whether terms in my research area are being used consistently across authors.	5-point scale
Q23	[C] Because terminology variation and ambiguity issues in phenotype descriptions are inevitable due to human nature, I do not think we can do anything to substantially improve the situation for any taxonomic group.	5-point scale
Q24	[B] I prefer the freedom to write my manuscript my way. As long as it is published, I do not care how it will be used or not used by others or computers.	5-point scale
Q26	[B] My current effort in using established terminology in my scientific writing can be best described as	^a^No effort—using established terminology never comes to mind; No effort—I believe my colleagues/readers understand my terminology well; Some effort—using established terminology is my intention; Significant effort—sometimes going out of my way to find the best terms to use;

a In Figure 11, the labels for these choices are simplified to ‘unconsidered’, ‘my pub is clear’, ‘intend to use’ and ‘used’, respectively.

In addition to the correlation analyses, structural equation modeling is used to confirm the relationship among respondents’ awareness of the ambiguity problem, their resistance to use controlled vocabularies/ontologies and their commitment to adopt potential solutions.

## Results

Qualtrics recorded 136 responses to Version 1 of the survey and 30 responses to Version 2. Among these 166 responses, 97 effectively completed the survey (i.e. completed 80% or more of the questions), while the other respondents completed the demographic information or less.

Upon examining the responses to the workload distribution question (Table 1, Q7), 6 of the 97 respondents reported ‘rarely’ work on phenotype data (i.e. the first three work types). Their data are therefore excluded from the subsequent analysis, making the effective total respondents 91. These respondents spent a median of 9 minutes (min = 3.8 min) answering the survey. The CSV files containing all the original 136 and 30 responses and the CSV files containing the effective responses are included in Supplementary Appendix A. The IP addresses and geo-location information have been removed from the CSV files to maintain the anonymity of the respondents.

We examined the data from the 69 respondents who opened the survey but did not answer any nondemographic questions. We failed to identify any patterns in the (demographic) data collected from these respondents. We observed that 80% of them who answered the ‘years of experience’ question (Table 1, Q5) had more than 15 years of experience. However, 50 out of 91 respondents who completed the survey also had longer than 15 years of experience.

**Figure 1.**
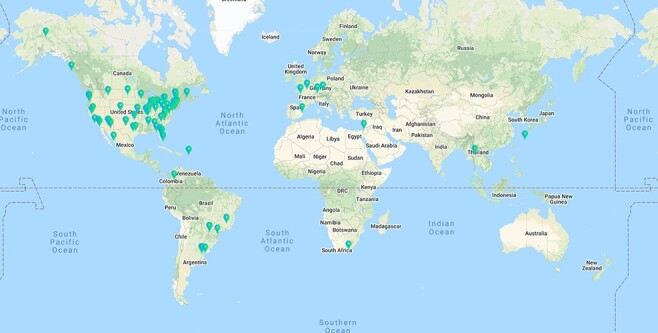
Geographical distribution of the 91 effective respondents. Geolocation resolution was conducted using https://ipapi.co/ on the respondent IP addresses collected by Qualtrics.

Among the 91 effective responses, 74 were from Version 1, and they formed the Biology Respondents group (*N* = 74). Of the 17 respondents from Version 2, 14 mainly worked in botany areas, and they formed the Botany Respondents group (*N* = 14). The geographical distribution of the 91 respondents is shown in Figure 1 using the geo-location resolution based on IP address.

Below, we report the descriptive results from the two groups for demographics and each of the research questions after presenting the related survey questions. These results are first reported separately for each of the group, and in the summary for each section, we report the summative results from the combined datasets covering all 91 respondents. We then report the results that show correlations and associations among different variables, along with a model constructed using the structural equation modeling method (16).

### Descriptive results

In each of the sections below, we first introduce the survey questions and then present the descriptive results. All questions except the demographic information questions are marked with [A] (for affective), [B] (for behavioral), [C] (for cognitive) or [T] (for an attitude) in the text, indicating the attitudinal component a question assesses.

#### Respondents’ demographic information

Table 1 lists the questions related to respondents’ demographic information. The question IDs (QID) are unique numbers used to refer to specific questions employed in the study, and they do not necessarily correspond to the order in which questions were asked during surveys. Q6v2.2 is one of the two questions added to Version 2 of the survey. Q2 was in Version 1 but not included in Version 2.

Demographic information helps us understand whose opinions we have collected and the representativeness of the samples who responded to the survey. The question on respondent’s job title (Version 1 only) accepted free text, and we received over 50 distinct job titles that were categorized into trainees (graduate students and post-docs, 9), tenured or tenure-track academics (21), research scientists (20) and collection curators or managers (24). Work performed under different job titles could overlap, and they are captured by Q7 and results presented in Figure 3.

**Figure 2.**
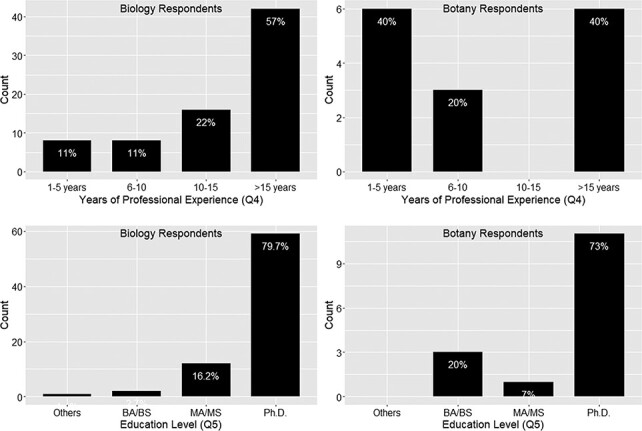
Years of professional experience (Q4) and education level (Q5).

**Figure 3.**
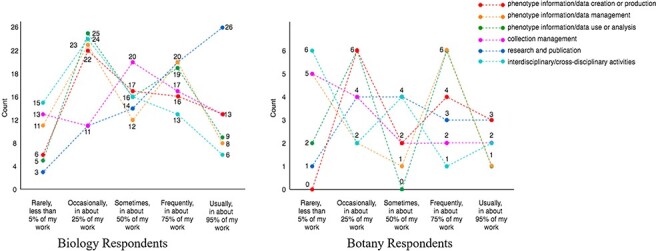
Respondents’ work distribution (Q7).

Figures 2 and 3 display the results (counts) of the answers to demographic questions.

Respondents’ workload distribution (ranging from ‘rarely, <5%’ scored as 1, to ‘>95%’ scored as 5) among different work types are summarized as a multiline plot in Figure 3. Each line represents a type of work, and the count (*y*-axis) is the number of responses. The counts marked on each line add up to the total responses for the work type.

Figure 3 shows that all respondents in the dataset cover at least one of the phenotype data-specific work types (i.e. the first three types in the figure) with 25% or more effort. Looking at the crossing patterns of the lines for the Biology and the Botany Respondents in Figure 3, we see that everyday work of the respondents of this survey covers a mixture of work types to varied degrees. Not shown in the graph, 79 of the 91 respondents’ workloads among the work types were distinct from others. This shows that, although the number of respondents is not large, the survey reached a variety of individuals who work extensively with phenotype data.

In summary, the 91 respondents of the surveys regularly work with phenotype data, and there is a stronger emphasis on research and publication and less so on cross-disciplinary/cross-taxon work. Eighty-four percent of respondents have more than 5 years of work experience, while 79% of respondents hold a doctorate.

#### Respondents’ current experience and overall attitudes toward controlled vocabulary

Table 2 lists the questions related to current experience and overall attitude toward controlled vocabularies. Q6v2.1 is one of the two questions added to Version 2, while Q8vI is the question in Version 1 but removed from Version 2.

**Figure 4.**
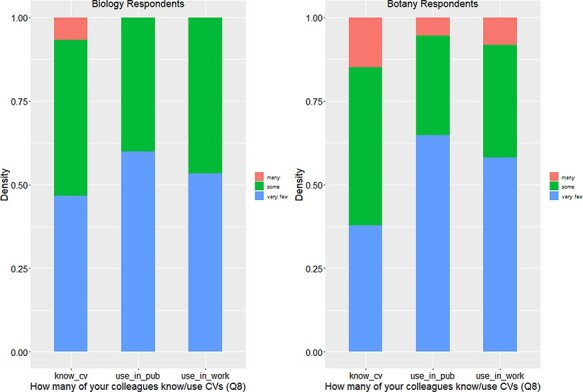
Number of colleagues who know or use controlled vocabularies (Q8).

**Figure 5.**
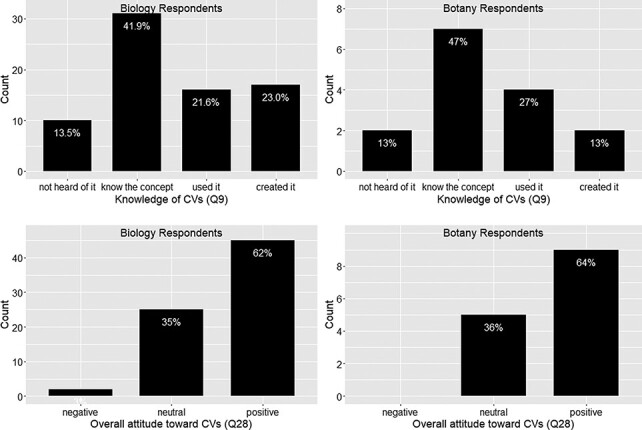
Knowledge of controlled vocabularies (Q9) and overall attitude toward controlled vocabularies (Q28).

Q3v2.1 was only included in Version 2, where 73% of the 17 respondents confirmed that they were familiar with the cited terms, suggesting Botany Respondents are equally aware of controlled vocabularies and the curation process as other respondents (see also Figures 4 and 5).

Q8vI was only included in Version 1. Of 74 respondents, 41 listed controlled vocabularies known to them. These controlled vocabularies can be grouped into: (i) glossaries and thesauri, such as Nomen, the Flora of North America Glossary and glossaries for different zoological groups; (ii) terminology set, e.g. Dublin Core and Darwin Core and (iii) formal ontologies hosted at OBO Foundry or BioPortal, such as Gene Ontology, Hymenoptera Anatomy Ontology, Phenotypic Quality Ontology and Plant Ontology. Roughly, the same numbers of zoology ontologies/glossaries were mentioned as plant ontologies/glossaries. This shows that Version 1 reached both plant and zoology communities; however, we observed that respondents either listed a set of formal ontologies or a set of glossaries/thesauri; very rarely did we see formal ontologies and glossaries listed together by one respondent.

Figures 4 and 5 show that responses from Botany Respondents and Biology Respondents are very similar on Q8, Q9 and Q28.

In summary, among 91 respondents, a small portion (13% or 14.1%) reported that they had never heard of controlled vocabulary, and about 20% of the respondents had experience creating a controlled vocabulary (Figure 5). No more than 12.5% of respondents work in controlled vocabulary circles (i.e. know many colleagues who know or use controlled vocabularies; Figure 4). While most respondents are aware of controlled vocabularies, a majority of respondents (over 50%) believe very few of their colleagues use controlled vocabularies in their publications or in their work (Figure 4). These findings suggest (i) the awareness of controlled vocabularies is widespread, (ii) packs of controlled vocabulary users exist but (iii) the actual use of controlled vocabularies in publications or in work is by no means a common practice. Despite a low overall integration of controlled vocabularies in publication/working practices, a solid majority (64%) of the respondents reported a positive attitude toward controlled vocabularies, while only 2% expressed an overall negative attitude (Figure 5).

#### To which degree respondents’ awareness of data quality and curation issues?

Questions listed in Table 4 assess respondents’ awareness of data quality and curation issues.

**Figure 6.**
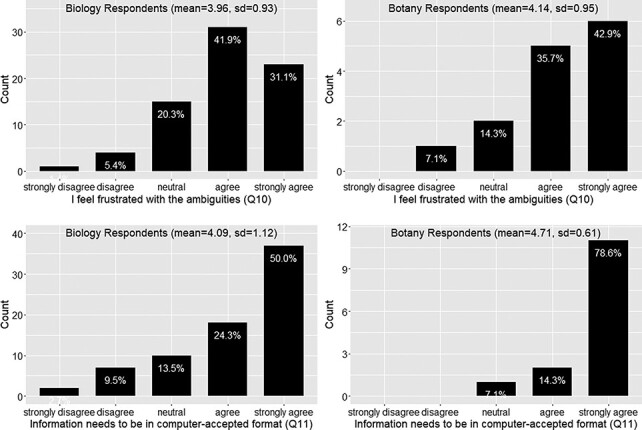
Frustration with ambiguities in phenotype descriptions (Q10) and position on the need for information to be in a computer-accepted format for computation” (Q11).

**Figure 7.**
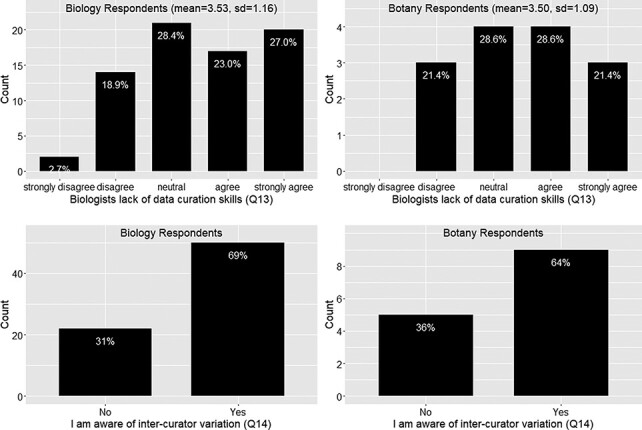
Lack of data curation skills (Q13) and inter-curator variation awareness (Q14).

**Figure 8.**
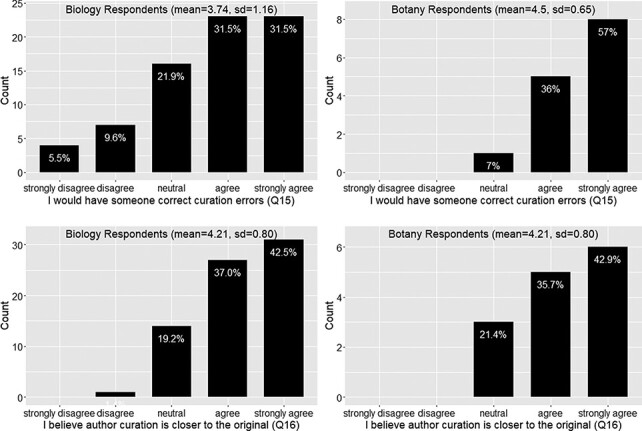
Action to correct curation errors (Q15) and position on whether authors or data curators are more capable of retaining the original meaning of a character (Q16).

Again the responses from the botany group follow the similar trend as the biology group on this set of questions. In summary, around 73% of the respondents felt frustration with the ambiguity in phenotypic publication, with a mean agreement score = 4 (agree) (Figure 6). About 78% of the respondents agreed that phenotypic information needs to be curated (converted to machine acceptable format) to support computation with a mean agreement score >4 (agree to strongly agree) (Figure 6). While curation is necessary, close to 50% of respondents agreed that biologists lack data curation skills, while 30% did not agree or disagree on this statement and 20% disagreed. With a mean agreement score of 3.5, there is not a clear agreement on this question (Figure 7). In terms of the issues involved in the curation process, around 67% of respondents were aware of inter-curation variation (Figure 7) and over 65% of respondents would make an effort to correct curation errors (Figure 8). However, with a mean agreement score of 3.87 and standard deviation of 1.12 with the latter, there does not seem to be a strong will to correct curation errors. At the same time, there is a strong agreement (around 80% of respondents agree with mean score = 4.21) that author curation would better reflect the original meaning of the phenotype data (Figure 8).

#### User’s attitude and current actions toward data quality issues?

**Figure 9.**
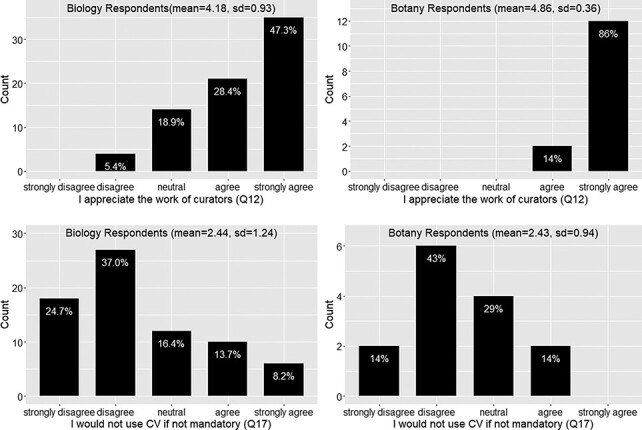
Appreciation of the work of curators (Q12) and willingness to use controlled vocabularies if not mandatory (Q17).

**Figure 10.**
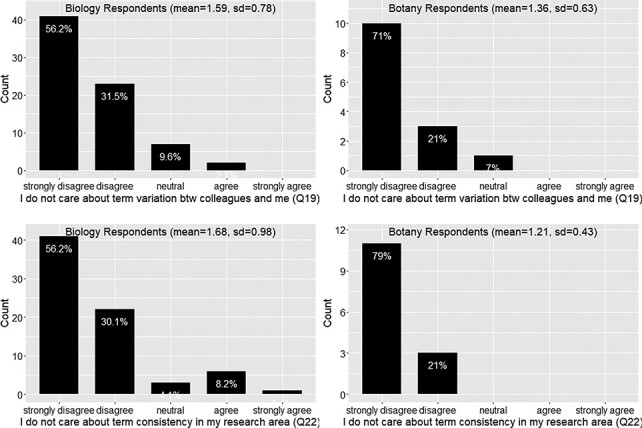
Care about term variation (Q19) and care about term consistency (Q22).

**Figure 11.**
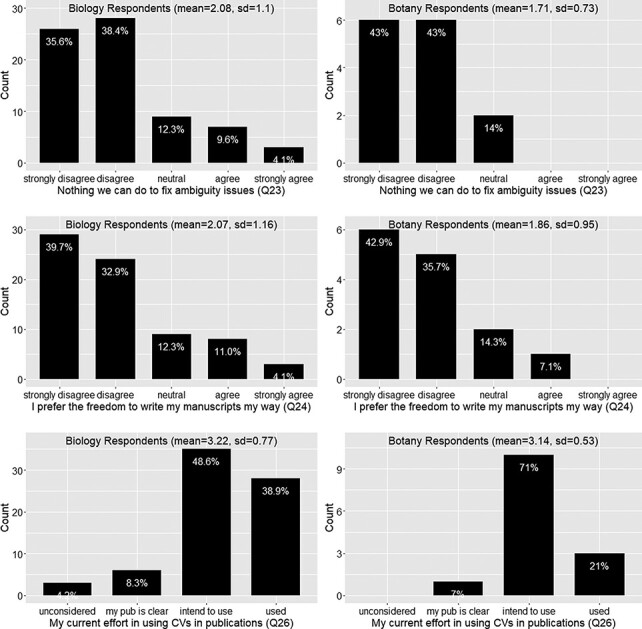
Ambiguity issue cannot be solved (Q23), preference for the freedom to write manuscript own way (Q24) and current effort in using controlled vocabularies in publications (Q26).

Figures 9–11 show a similar pattern between the responses provided by the Biology and Botany groups. In summary, there is a solid appreciation of data curators’ work (Figure 9) and a clear concern about data consistency and clarity in publications (Figure 10). Respondents would not put personal freedom above data quality (agreement score = 2, meaning they disagree with the question statement; Figure 11) and believe work can be done to address the ambiguity issues (agreement score = 2; Figure 11). Further, the mean current effort score is greater than 3, which corresponds to ‘intend to use controlled vocabularies’, an answer selected by over 88% of respondents. We were pleased to see that there were about 33% of respondents already using controlled vocabularies in their publications (Figure 11).

**Figure 12.**
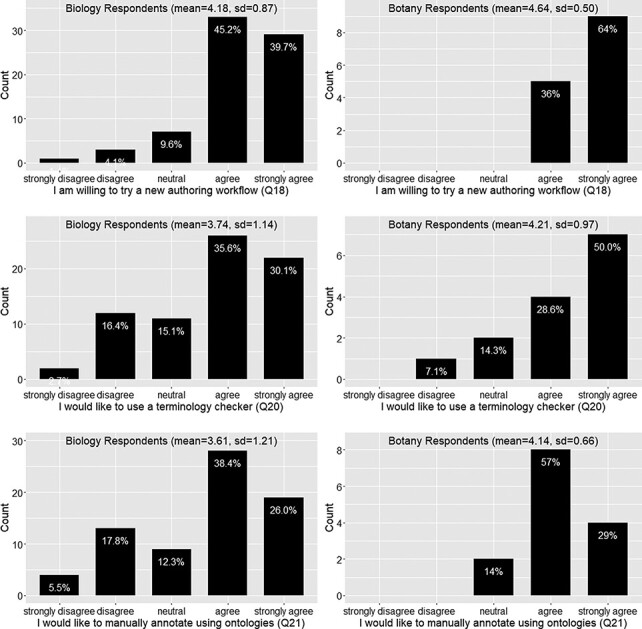
Willingness to use a new authoring workflow (Q18), a terminology checker (Q20), or ontologies to manually annotate scientific writing (Q21).

#### Respondents’ preference toward candidate solutions

The ultimate goal of the project ‘Authors in the driver’s seat: fast, consistent, computable phenotype data and ontology production’ is to investigate an approach to addressing the data quality issues that is different from the existing data curation model anchored on the curators. Therefore, it is important and necessary to be aware of people’s preferences toward a solution.

Table 5 presents the questions related to respondents’ preferences toward a solution.

**Table 5.** Questions related to preferences toward a solution

QID	Questions	Options
Q18	[B] I am willing to try a new workflow to record phenotypic characters (e.g. measurements) if that helps improve the consistency and usefulness of phenotype information across my community.	5-point scale
Q20	[B] I would like a terminology checker software (like a spelling checker) to check how well my scientific writings use established terminologies (semi)automatically.	5-point scale
Q21	[B] I would like to use an ontology to manually annotate my scientific writing using established terminologies (e.g. annotate ‘stout’ in my text as a formal term ‘increased size’).	5-point scale
Q29	My suggestions to make the production of computable phenotypic characters more effective than human curation:	Text input box

Figure 12 shows that a vast majority of the 91 respondents (87.8%) would like to try a new authoring workflow to make the data more consistent and reduce ambiguity. The mean agreement scores are greater than 4 for both Botany and Biology Respondents. Botany Respondents seem to agree more strongly than the Biology Respondents on their willingness to try a terminology checker or to try a manual annotation approach using an ontology; however, Mann-Whitney U tests did not find that the two distributions were different (*P* = 0.2 and *P* = 0.14, respectively). A Spearman’s rank correlation test performed on the entire dataset found that the ranks for Q20 and Q21 are relatively strongly correlated (rho = 0.66, *P* < 0.001), suggesting that respondents favoring one approach also favor the other approach. While there is not a solid agreement on what new authoring workflow respondents prefer (overall agreement score < 4; see also Figure 12), the result does show that over 50% of the respondents were willing to use ontologies for manual annotation. This may be a useful finding for scientific journal publishers who are considering whether they should ask authors to perform a level of annotation on their manuscripts.

Q29 was an open-ended question that asked the respondents what they think needs to be done to address data quality issues. A total of 40 answers were collected. The most prominent themes we identified from the responses are listed below:

1. Make it easy so it is understandable and accessible by all authors and benefits the authors.
2. Automate curation processes but expose the steps to users who care to know (not make the processes a ‘black box’).
3. Make ontologies taxon specific, flexible and not distort the original meanings of the term to fit the complex structure of an ontology.
4. Educate a wide range of users, from biology students to authors, editors, reviewers and journal publishers.
5. Make using controlled vocabularies a requirement for journal publication.

#### Respondents’ claimed effort and desired reward

To design an effective solution that users would adopt, we asked two questions to understand the expected effort users are willing to commit and the rewards for adoption that are desirable by the users. These questions are shown in Table 6.

**Table 6.** Questions related to committed effort and desired rewards

QID	Questions	Options
Q25	[B] The additional effort I am willing to put (e.g. in using a terminology checker) to make my manuscripts more accessible to computation and to scientists within and outside of my discipline is:	5-point scale
Q27	If I make an effort to make my phenotypic characters more useful for computation and others’ research, my preferences for potential rewards are: (1) Citations—Make the terms I added to a controlled vocabulary (to share with my community) citable (2) Have computer convert my character information to other useful formats (e.g. tabulate the characters for me) (3) Have computer format my character/measurement descriptions for publication (4) Citations—Make my character data citable (5) Monetary rewards	Ranks from 1–5: 1 = most preferred 5 = least preferred

**Figure 13.**
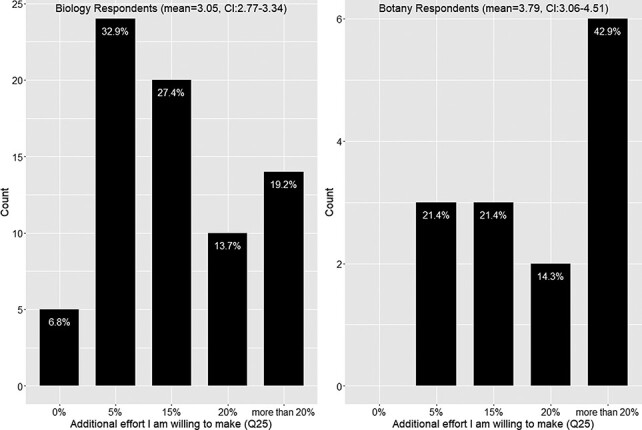
Additional effort respondents are willing to put into making manuscripts more accessible to computation (Q25).

A Mann-Whitney U test found a nearly significant difference in the distributions of efforts selected between the Biology Respondents and the Botany Respondents (*P* = 0.055). From the data shown in Figure 13, the distribution of the Biology Respondents is skewed toward the left, while the distribution of the Botany Respondents is skewed toward the right. Botany Respondents on average are willing to endure a 15% effort resulting from adopting a new authoring workflow (95% confidence interval = [3.06–4.51], where 3 indicates 15% additional effort) with more than 50% respondents claiming 20% or more additional effort. In contrast, the Biology Respondents on average seem to like the additional effort to be between 5% and 15%. (95% confidence interval = [2.77–3.34], where 2 indicates 5% additional effort).

**Figure 14.**
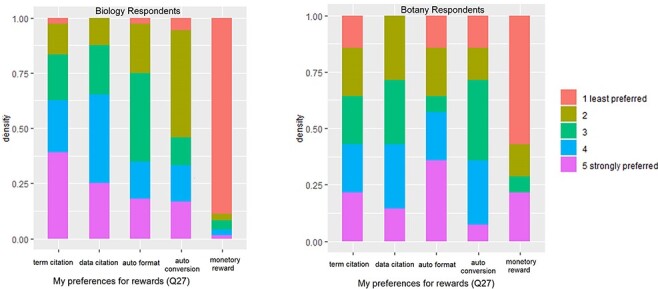
Preferences for rewards (Q27).

Q27 asked the respondents to rank their preferences over a set of rewards that would compensate them for the additional effort they would need to invest toward adopting and using a new authoring workflow. The results are shown in Figure 14. For each award type, a Mann-Whitney U test was performed to detect any difference between the Botany Respondents and the Biology Respondents. Only the monetary awards showed a significant difference (*P* = 0.003), which is also visible in Figure 14.

Given no statistically significant difference in the other four reward types between Botany and Biology Respondents, we used the combined data to rank the overall preferences toward the reward types and included the results in Table 7. Results from the Mann-Whitney U tests show that the preferences toward the two citation types were the strongest, followed by Auto Format and then by Auto Conversion, and Monetary Rewards were the least preferred. The differences among the four levels of preference were statistically significant (*P* < 0.05; Table 7).

**Table 7.** Ranking of respondents preferences toward five different reward types (*N* = 88). The lower the mean the stronger the preference

Q27 If I make an effort to make my phenotypic characters more useful for computation and others’ research, my preferences for potential rewards are (1 = most preferred, 5 = least preferred)	1	2	3	4	5	Mean (standard deviation)	Preference Level (*P* < 0.05)
[Data Citation] Citations—Make my character data citable	22	33	20	13	0	2.27 (1.00)	1
[Term Citation] Citations—Make the terms I added to a controlled vocabulary (to share with my community) citable	31	21	18	14	4	2.31 (1.23)	1
[Auto Format] Have computer format my character/measurement descriptions for publication	18	16	31	19	4	2.72 (1.15)	2
[Auto Conversion] Have computer convert my character information to other useful formats (e.g. tabulate the characters for me)	13	16	15	38	6	3.09 (1.21)	3
[Monetary Rewards] Monetary rewards	4	2	4	4	74	4.6 (1.01)	4

### Correlation and association results

As shown above, the responses from the Botany Respondents and Biology Respondents are quite similar. In this section, we will use the 91-response dataset to answer a set of questions on how variables are related.

(i) Are demographic variables (Q4, Q5, and Q7) correlated with respondents’ knowledge about controlled vocabulary (Q9), controlled vocabulary usage practices (Q26), attitude toward controlled vocabulary (Q28), willingness to adopt a new authoring workload (Q18) or claimed additional effort (Q25)?

Spearman tests were conducted between other demographic variables and the above-mentioned attitude variables (Q9, Q26, Q28, Q18 and Q25).

With 95% confidence, length of work experience (Q4) was found to weakly but significantly correlate only with knowledge about controlled vocabulary (Q9, Spearman, rho = 0.23, *P* = 0.03). This result does not support the claim that younger researchers embraced controlled vocabulary more or are more willing to adopt new methods.

Education level (Q5) was found to be negatively corrected with claimed additional effort (Q25) with rho = −0.26, *P* = 0.01, suggesting the higher the education level, the lower extra effort one is willing to make.

Work types involving phenotype information/data creation and management (Q7 Type 1 and Type 2) were found to be positively correlated with claimed additional effort with rho = 0.22, *P* = 0.03 and rho = 0.28, *P* = 0.006, respectively. This suggests the more deeply respondents are involved with phenotype data creation and management, the more effort they are willing to invest in addressing data quality issues. This finding is encouraging. Additionally, Q7 Type 2 (data management) is correlated with controlled vocabulary usage practices (Q26, rho = 0.32, *P* = 0.002), suggesting respondents deeply involved with data management use controlled vocabulary more. This finding reflects the reality. However, no significant correlation was found between any work type and frustration with ambiguity (Q10), suggesting the level of frustration (Figure 6) that is shared across the work types.

(ii) How are affective, cognitive and behavior variables related to one another and to the overall attitude toward controlled vocabularies and the adoption of a new workflow?

**Figure 15.**
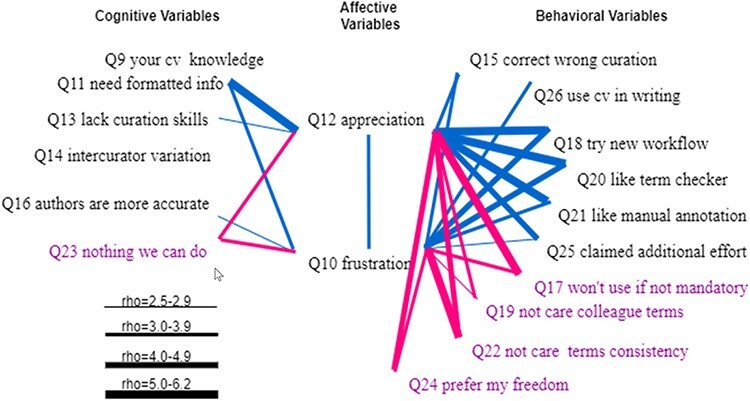
Spearman correlations between affective variables (Q10 and Q12) and (1) cognitive variables (Q9, Q11, Q13, Q14, Q16 and Q23) and (2) behavioral variables (Q15, Q26, Q18, Q20, Q21, Q25, Q17, Q19, Q22 and Q24). Variables that indicate a resistance to change are displayed in purple. All links represent statistically significant correlations. Blue links represent positive correlations, while red links for negative correlations. The thickness of the links indicates the strength of a correlation.

Figure 15 shows the statistically significant Spearman correlations between the two affective variables and cognitive and behavioral variables. The two affective variables, Q12 and Q10, were correlated with rho = 0.38, and the two variables had a rather similar profile of correlations with the six cognitive variables and the 10 behavioral variables, with a few exceptions. The more the respondents agreed that computers needed formatted information, the more they appreciated curators’ work, and the more they felt frustrated with the existing ambiguity. In contrast, the more the respondents agreed that there was nothing we could do to solve the ambiguity issues, the less they appreciated curators’ work, and the less they felt frustrated. At a weaker but detectable level, we found (i) the more the respondents believed that authors lacked curation skills, the more appreciation they had for curators, and (ii) the more frustrated the respondents were, the more they believed author curation would be more accurate.

We note that respondents’ controlled vocabulary knowledge and whether they were aware of inter-curator variation were not correlated with their frustration or appreciation levels.

However, both affective variables were correlated with almost all behavioral variables with one exception (that Q12 and Q26 were not correlated). Affective variables were positively correlated with variables indicating a will to act and negatively correlated with variables indicating a resistance to changes.

**Figure 16.**
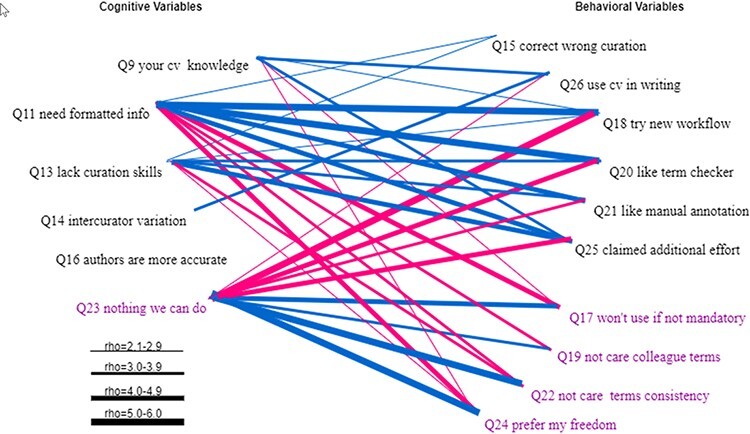
Statistically significant Spearman correlations between the cognitive variables and the behavioral variables. Variables that indicate a resistance to change are displayed in purple. All links represent statistically significant correlations. Blue links represent positive correlations, while red links for negative correlations. The thickness of the links indicates the strength of a correlation.

Statistically significant Spearman correlations between the cognitive variables and the behavioral variables are displayed in Figure 16. The general pattern is that the resistance cognitive variable (Q23) is positively correlated with the resistance behavioral variables (Q17, Q19, Q22 and Q24) and negatively correlated with the action behavioral variables (Q15, Q26, Q18, Q20, Q21 and Q25). Other cognitive variables are positively correlated with the action behavioral variables and negatively correlated with the resistance behavioral variables.

A notable finding is that Q16 was not found to be correlated with any of the behavioral variables, indicating that respondents taking or resisting actions to solve the ambiguity issues equally believed that authors are more accurate (also see Figure 8).

How do the cognitive factors influence the behavior of the respondents (take actions vs. resist to change)? Figure 16 shows clearly that cognitive variables Q11, Q23 and Q13 have the strongest correlations with the behavioral variables (especially Q18 and Q25) and therefore have the greatest influence. We had expected that respondents’ knowledge of controlled vocabulary (Q9) would have the strongest impact on behavior, but the data suggests that the awareness of the need to format data for computers has the strongest impact. This makes sense because ontologizing phenotype data is only one of many different ways to format the data.

(iii) How do cognitive variables and behavioral variables correlate with other variables in the same group?

We found statistically significant correlations using Spearman tests among almost every pair of the behavioral variables (except Q26), with the rho ranging either from 2.1 to 7.0 (between Q20 and Q18) among action variables (shown in black in Figure 16) and among resistance variables (shown in purple in Figure 16) or from −2.1 to −5.6 (between Q24 and Q18) between an action variable and a resistance variable. This dense and dichotomous correlation pattern in the behavioral variables and between behavioral and cognitive variables shown in Figure 16 indicate a clear separation between respondents who would take actions to improve data quality and those who resist changes.

Unlike other variables, Q26 was only correlated with Q17, Q22 and Q25. These correlations are easy to explain; however, we would point out the variables with which Q26 lacks correlations: Q18, Q20 and Q21. This suggests that whether respondents are currently using controlled vocabularies in their writings, they were equally likely to try a new workflow. Another interesting observation is that the correlation between Q17 and Q20 was −0.32, while the correlation between Q17 and Q21 jumped to −0.52, suggesting that respondents who would not use controlled vocabularies unless required dislike manually curating their writings more than they dislike using a term checker.

**Figure 17.**
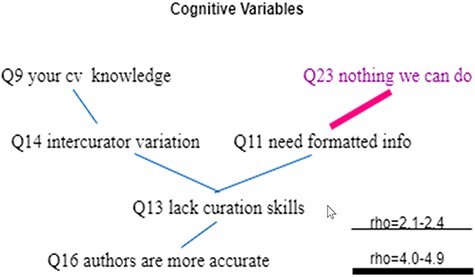
Statistically significant Spearman correlations among cognitive variables. Variables that indicate a resistance to change are displayed in purple. All links represent statistically significant correlations. Blue links represent positive correlations, while red links represent negative correlations. The thickness of the links indicates the strength of a correlation.

The correlations among the cognitive variables were less dense, with one variable correlating with no more than three other variables (Figure 17). Interesting findings here include:

1. Q23 was only correlated with Q11, with a relatively strong rho of −0.45. In other words, other knowledge (e.g. controlled vocabularies) or beliefs (e.g. biologists’ lack of curation skills) has no (linear) impact on Q23. And Q11 was only correlated with Q13.
2. Q13 and Q16 are weakly positively correlated, suggesting that despite holding the belief that authors lack curation skills, these respondents also tend to believe that authors would be more accurate in converting phenotype information into a computer format.

### Structural equation modeling result

Based on the findings above, we hypothesized that the awareness of the challenges for computable phenotype data (Problem or PRO) would impact respondents’ acceptance of potential solutions (SOL) and their possible resistance to changes (RES). We conducted a confirmatory factor analysis using the lavaan package in R (16) to test whether the data fit this hypothesized measurement model. We have a total of 91 observations, which is the acceptable size to test the fit of a three-latent-factor model, where each factor is represented by two variables. Such a model has 15 free parameters to estimate, making the observation (91) and parameter (15) ratio greater than 5, a recommended lower bound for the observation size (17).

**Figure 18.**
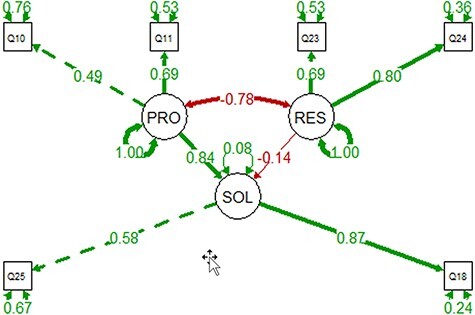
The PRO-SOL-RES model constructed with the 91 observations. Circles are for latent variables, and boxes are for measured variables. Self-directing, double-arrowed links show the residuals. Double-arrowed links between two variables indicate correlation, where single-arrowed links show causal relationships. Positive correlations are shown in green, and negative in red. The model shows that increased awareness of the problems can reduce resistance and increase adoption.

For the latent factor PRO, we selected Q10 [A] and Q11 [C] from Table 3 that holds questions assessing respondents’ awareness of the problems. These two variables are also shown in Figures 15–17 as the variables that correlate with the largest number of the behavioral variables. For the latent factor SOL, the most relevant variables are Q18[B] (Table 5, willingness to use a new workflow) and Q25[B] (Table 6, claimed additional effort), and both are shown in Figures 15 and 16 as the variables that correlate with the largest number of the cognitive and affective variables. For the latent factor RES, we selected Q23[C] and Q24[B] as they correlate with the largest number of other variables. The model and correlations among latent factors are shown in Figure 18. The model is confirmed by lavaan to fit the data very well: both Comparative Fit Index (CFI) and Tucker-Lewis Index were 1.00 (CFI >0.95 is considered good fit), RMSEA = 0 and *P*-value for RMSEA ≤0.05 is 0.652 (RMSEA <0.05 is considered good fit); all variances were greater than 0 and *R*^2^ for SOL was 0.92, indicating the data fits the model well, the variables predict SOL very well with small residuals (*R*^2^ = 1 for a perfect fit); and all loadings (numbers on the single-arrowed links) of measured variables were above 0.3.

The model shows that increased awareness of the problems would reduce resistance and increase adoption of the solution. Similarly, increased resistance would result in reduced adoption. More importantly, problem awareness has much greater impact on solution adoption than resistance does (betas of the regression = 0.84 vs. 0.14).

## Discussion

### Survey results

Our results provide a better understanding of the attitudes biologists have toward the use of controlled vocabularies/ontologies as a potential strategy to ensure that published phenotype data are computable. Based on the data and the analysis, we present the answers to the five research questions as follows:

(i) What are the respondents’ current experience and overall attitude with controlled vocabularies?

Of the respondents surveyed, 60% held a positive attitude toward controlled vocabularies, and 37% were neutral (Q28; Figure 5). Although 87% of respondents were aware of controlled vocabularies, most have no experience using or creating them (Q9; Figure 5). In fact, 60% of the respondents had never used a controlled vocabulary in their publications, although many plan to do so. It is worth noting that 13% of respondents had never heard of controlled vocabularies (Q26; Figure 11). Because there is a positive correlation between the knowledge of controlled vocabularies and their use in scientific writing (Figure 16), to encourage authors to use controlled vocabularies, we need to reach out to these groups (e.g. through workshops) so that all phenotype data workers become knowledgeable about different types of controlled vocabularies and their benefits. Providing tailored workshops was one of the suggestions made by the respondents for Q29.

(ii) What is the respondents’ awareness of data quality and curation issues?

Three quarters of respondents agreed or strongly agreed that phenotype information needs to be formatted in a way for computational analyses (Q11; Figure 6). While professional curation is one way to achieve this, data quality is adversely affected owing to ambiguity in published character descriptions, a fact that frustrated 70% of respondents (Q10; Figure 6), and problems with substantial inter-curator variation, of which 70% of the respondents were aware (Q14; Figure 7). The vast majority (80%) of respondents agreed or strongly agreed that the authors themselves should produce a more accurate curation of their own work (Q16; Figure 8), but 50% of respondents believed that authors lacked the necessary curation skills and software tools to do the job. This highlights the fact that creating easy to use and intuitive software, which will facilitate author curation, is a critical step toward making pre-publication author curation a reality.

(iii) What are the respondents’ attitude and current actions toward data quality issues?

A strong desire exists amongst respondents to improve data quality:

- A majority (76%) of respondents appreciate curators’ work (Q12; Figure 9)
- A large majority (88%) of respondents care about the consistency in terminology usage in their fields and among their colleagues (Q19 and Q22; Figure 10)
- A majority (74%) of respondents disagree that there is nothing we can do to solve the data quality issues (Q23; Figure 11)
- A majority (72%) of respondents disagree that they prefer the freedom to write their characters their way (Q24; Figure 11)

However, it is also important to note that less than 40% of respondents were currently using a controlled vocabulary (which includes a nomenclature, Q26, F18) and about 23% of the respondents will not use a controlled vocabulary unless it is mandatory (Q17, Figure 9). In the next section, we discuss the elements that would be helpful to turn the desire of majority phenotype data workers into real actions to proactively address the data quality issues and the elements that are needed to bring the reluctant into this effort.

(4 and 5) What is the respondents’ preference toward a solution? What are the respondents’ claimed additional effort and desired rewards?

The vast majority (87.8%) of respondents were willing to try a new authoring workflow to publish FAIR phenotype data (Q18; Figure 12), but only about 65% of the respondents would like to use an automated terminology checker (Q20; Figure 12). Likewise, 65% of respondents agreed they would like to use ontologies to manually curate their own writing (Q21; Figure 12). As noted above, these two options were not competing but correlated, and respondents who refused to use a controlled vocabulary unless it was mandatory disliked manual annotation more than using a term checker. Respondents also stated that a ‘black box’ solution is not desirable (Q29). In addition, our survey indicates that almost all respondents (93%) would adopt a new workflow if it required only 5% of additional effort to use as compared to their existing workflow. However, the adoption rate would fall dramatically to 60% if an additional effort of 15% was required to adopt a new workflow, and only a minority of respondents (30%) would adopt a new workflow if it required an additional 20% of effort (Q25, Figure 13).

Given these facts, we have a set of rough requirements for the software: the software platform should support a workflow that translates potentially complex curation steps into tasks that are solvable by authors in a transparent, efficient and nonconfusing way. Sharing curation results among authors is a promising way to promote consistency and increase efficiency simultaneously (18). To further incentivize the adoption of the new workflow, it needs to support term citation and data citations, as these are the top rewards preferred by authors (Q27, Figure 14). It is also desirable for the platform to automatically format author-curated data into a format suitable for publication and to automatically convert author-curated data into other machine consumable formats.

From the Results presented above, there appears to be a dichotomy between authors willing to start creating and publishing FAIR data and authors who would strongly avoid using a controlled vocabulary if given the choice. Although the second group represents a minority of authors (Figures 10–12), it is important to note that using controlled vocabularies in publications is not popular practice (Figure 11) and the vast majority of publishing venues do not require authors to use controlled vocabularies. Understanding why authors are reluctant to use controlled vocabularies is important as we proceed with our research. The development of an intuitive user interface and demonstrating to authors the benefits of using software that facilitates the standardization of phenotype descriptions are important first steps.

The model shown in Figure 18 suggests several avenues that could increase the chance that controlled vocabularies would be adopted for standard use when creating phenotype data solution (spending additional effort (Q25) to use a new authoring workflow (Q18)):

1. Increase the awareness of the ambiguity problems associated with free-text phenotypic data. This will affect Q11 and Q10 positively and lead to increased adoption of controlled vocabularies and a new workflow. Authors may not be aware of the ambiguity in their publication and how it affects data aggregation, formatting and use in downstream computational analyses. Our results suggest that if they did, it would help motivate their adoption of a solution. Results (Q11; Figure 6) show that around a quarter of respondents were neutral or disagree that phenotype information needs to be in a computer-accepted format to be widely useful. We need to reach out to the authors sharing this view.
2. Another approach suggested by Figure 18 is to reduce resistance to the use of controlled vocabularies, which is highly correlated with the belief that there is ‘nothing we can do’ (Q23) and the preference for ‘freedom’ (Q24) when creating phenotypic descriptions. Figure 17 shows that responses to Q11 negatively correlate with Q23. Figure 16 shows Q12 negatively correlated with Q24, which suggests that when people appreciate the hard work of curators more, they better understand how sometimes one’s freedom could cause serious problems for others. When user-friendly workflows are adopted by authors, they will have a positive first-hand experience in curation. Furthermore, when positive progress is made to solve the problem (e.g. user-friendly software platform, curated data) these tools need to be made publicly available and broadly advertised to demonstrate that many issues such as ambiguity in phenotypic descriptions can be addressed when a controlled vocabulary is used.

### New authoring workflows

To make future published phenotype articles more computable, we believe the new authoring tools must support the production of both human-readable contents and the corresponding machine-actionable data at the same time, with persistent links between the two. The machine-actionable data may consist of a few important facts presented in the human-readable version, e.g. main conclusions in a medical article, such as ‘Moderna COVID-19 vaccine reduces serious COVID-19 cases’. It may also contain all the data, such as a complete taxon-by-character matrix underlying a taxonomic treatment. Such data will likely be expressed as one or a group of interconnected RDF (Resource Description Framework) graphs using relevant ontologies. These RDF graphs and the human-readable content can reside on the same or different servers but permanently connected with persistent IDs (PIDs, e.g. DOI states PID_of_a_fact). With the persistent IDs and relevant ontologies, the RDF data, curated by the author, can be converted in any desired format and used directly in computation, bypassing the data curation step altogether. For example, using good PIDs and ontologies, multiple RDF graphs can be easily put together to create a linked open data cloud for a specific domain or merged into The Linked Open Data Cloud (https://www.lod-cloud.net/). Anyone can query the cloud to get computable phenotype data relevant to their research questions (e.g. in life sciences (19)). In essence, the RDF graph(s) is an alternative computable representation of the knowledge published in a human-readable article.

The survey results indicated that a small number of authors are already using controlled vocabularies in their work or publications. This practice should be commended, but we should also point out the difference between a manuscript produced by a new authoring tool described above and a manuscript written in MS Word using terms from an ontology. The key difference is that the content of the former has a corresponding representation that is computable, while the latter will still have to go through some error-prone process of curation (e.g. text mining, ontology term matching and human validation steps) to produce computable data.

Authoring tools for taxonomists, such as DELTA (DEscription Language for TAxonomy) (20), Lucid key (21) and XPER^3^ (22), were created to improve the structuredness in taxonomic works, such as taxonomic descriptions or dichotomous organism identification keys. Although being complied with TDWG’s 2005 standard for Structured Descriptive Data (SDD) (23), they have not provided support for authors to use terms from phenotype ontologies to formulate their phenotype characters and values (i.e. states). SDD enables users to define their terminologies for characters, character states, modifiers, taxon concepts, etc. before these are used in the content of a description or a key. It is the users’ choice to use their locally defined terminology, or to select relevant terms from existing ontologies, which can be a daunting task even for experienced ontology engineers with domain knowledge. At the establishment of the SDD standard, not many formal ontologies exist, and the freedom of defining one’s own terminology probably has encouraged the adoption of the standard. However, the phenotype data produced with these SDD-complied authoring tools remains noncomputable. We believe it is time for the next round of extension of SDD to incorporate ontologies into the core of the standard.

Many may wonder why the above-mentioned feature-rich tools have not replaced Excel for many taxonomists, considering DELTA has been in use since 1970s. We observe that, on one hand, Excel still fulfills the needs of many taxonomists today when it comes to completing their taxonomic works. And on the other hand, few formal studies have investigated time savings or quality improvements these tools enable. In addition, many taxonomists have built a complex data infrastructure in Excel that cannot be easily imported into these systems. More importantly, publishing venues do not require taxonomic works to be in a structured format. Any taxonomic description or identification key of good quality for human readers is publishable regardless of the tools used to produce them. The time is different now. More and more authors feel the negative impact of not having computable data to use when large computational infrastructures have become available. These observations were the motivating factors behind the two surveys reported here and the usability studies we have been conducting on the software prototypes we are developing (e.g. (18)).

For new, ontology-aware, authoring tools to be widely adopted, we believe that: (i) publishing venues should require authors of phenotypic works (e.g. taxonomic descriptions) to provide some computable data for publication, as we now know at least 22% of authors will not use a controlled vocabulary if it is not mandatory; (ii) authoring tools with intuitive interfaces must be made available to support authors in producing computable data while they write descriptions/articles for human readers; (iii) support for nanopublications (24) needs to be incorporated in such software so authors will be rewarded with future citations for any piece of computable data they produce and, lastly, (iv) a smooth learning curve should be put in place to bring authors up to speed over a period of time: from using controlled vocabularies to using ontologies, from annotating phrases to annotating facts and from annotating simple facts to annotating more involved facts. The authoring tools cannot require a user to study a thick manual to learn or to demand dramatically higher effort than current tools (like Excel) because we now know that any extra effort greater than 5% of normal may reduce the adoption rate by 30% (Figure 13). The authoring interface would either translate human-readable content to candidate statements for the author to select or facilitate the composition of such statements by the author. It should support sharing and reusing phenotypes to encourage convergence and improve work efficiency. The authoring interface must also give authors control in terms of how they would like to express their data and allow the authors to add terms to the ontologies with ease (in (18), we argued that the Semantic Web technology is meant to support diverse views). The user interface should be very carefully designed and tested so that it facilitates the needs of authors to express themselves while directing complicated ontological phenomena to ontology engineers.

## Conclusion

Based on the 91 effective responses from biodiversity professionals who create phenotype data, manage it and use it as a significant part of their daily work, we have identified strong evidence that most authors are ready to adopt a new workflow to produce FAIR data at the time of publication. Factors that would help accelerate this process are identified and to an extent quantified. These factors include a user-friendly and efficient software-authoring workflow, an increased awareness of how text ambiguity makes formatting phenotype data for computational analysis extremely challenging and an increased understanding of different controlled vocabularies and their benefits for improving data quality. In addition, a set of author-preferred characteristics for this new authoring workflow are identified, including reward mechanisms that would boost its eventual adoption.

## Supplementary Material

baac001_Supp
