# Supplementary material for: Authors’ attitude toward adopting a new workflow to improve the computability of phenotype publications
Source: Database (Oxford). 2022 Feb 2;2022:baac001. doi: 10.1093/database/baac001 (PMC9278328; doi:10.1093/database/baac001)
Supplement: baac001_Supp [file baac001_supp.zip › Appendix B - Background and Terminology.docx]

**Background and Terminology in Version 1**

In order to answer larger biological questions, knowledge produced by different biology branches needs to be integrated. For that purpose, a large number of ontologies have been developed (e.g., see OBO Foundry). Ontologies hold established biological terms and the relationships among these terms, and they can be seen as an advanced form of controlled vocabularies. These ontologies are being used in a process called "data curation" to convert published phenotype knowledge (including taxonomic descriptions, character descriptions) from their current narrative format to a structured formal（i.e. computable format) that can be used in large scale computation. Data curators translate author’s terminologies as used in publications to the standardized terms in the ontologies

**Background and Terminology in Version 2**

A lot of trait information has been published, but it remains time consuming to compile and use published information, because the terminology used in the descriptions are often vaguely defined. For example, "skull-roof weak", "culms weak", and "blade midrib weak" all use "weak", but each of them has a different meaning.

In order to answer larger biological questions, trait information produced by different biology branches needs to be integrated. For that purpose, a large number of ontologies have been developed (e.g., see OBO Foundry). **Ontologies,** holding established terms and relationships among these terms, can be seen as an advanced form of **controlled vocabularies/glossaries**.

One way to make use of the published trait data is for **data curators** to use these ontologies to manually convert published trait/phenotype knowledge (including taxonomic descriptions, character descriptions, etc.) from their current narrative format to a structured format（i.e. **computable format**)  that can be used in large scale computation. In this process, data **curators** translate author’s terminologies used in publications to the standardized terms in the ontologies to reduce **ambiguity,** for example, "weak = poorly developed" in 'skull-roof weak', "weak=Inconspicuous" in "blade midrib weak".
